# Mild hydrostatic pressure preconditioning improves post-thaw motility of cryopreserved stallion semen

**DOI:** 10.3389/fvets.2026.1937911

**Published:** 2026-09-24

**Authors:** Eszter Losonczi, András Horváth, Csilla Budai, Katalin Szabó, Judit Cserepes, Zsolt Lang, Csaba Pribenszky

**Affiliations:** 1 Department of Animal Hygiene, Herd Health and Mobile Clinic, University of Veterinary Medicine, Budapest, Hungary; 2 Department of Obstetrics and Food Animal Medicine Clinic, University of Veterinary Medicine, Budapest, Hungary; 3 Institute of Animal Husbandry, Faculty of Agricultural and Food Sciences and Environmental Management, University of Debrecen, Debrecen, Hungary; 4 Dunamenti Reproductive Centre, Kútvölgyi Institute, Budapest, Hungary; 5 Medicontur Medical Engineering Ltd., Budapest, Hungary; 6 Department of Biostatistics, University of Veterinary Medicine, Budapest, Hungary

**Keywords:** CASA, cryopreservation, hydrostatic pressure, mild stress preconditioning, sperm motility, stallion semen

## Abstract

The success of stallion semen cryopreservation is limited by substantial variation among stallions and by temporal variation within individual donors. However, the equine breeding industry requires predictable, stable, and efficient cryopreservation protocols to support the increasing use and dissemination of valuable genetics. One possible strategy for improving semen freezing is to introduce a preconditioning step before cryopreservation to prepare spermatozoa for the unavoidable stresses associated with freezing and thawing. This study evaluated the effects of mild hydrostatic pressure preconditioning on semen samples from Arabian and Shagya-Arabian stallions.

Sixty-three semen samples from ten stallions were included in the study, spanning between July and April, in 31 collection days. Before routine cryopreservation, each sample was split into one control and six treatment aliquots, subjected to one of six hydrostatic pressure preconditioning treatments or maintained as control. After freezing and thawing, total motility (TM) and progressive motility (PM) were assessed using computer-assisted sperm analyser. Five of the six preconditioning treatments significantly improved both TM and PM compared with the control (*p* < 0.05). TM increased from 51.87% in control samples to 56.39–59.17% in mild-hydrostatic-pressure-treated samples, while PM increased from 39.56% to 43.81–47.00%.

In our study, mild hydrostatic pressure preconditioning was readily integrated into the routine semen cryopreservation workflow and improved post-thaw motility. These findings indicate improved motility-based cryosurvival of stallion spermatozoa; however, the study did not assess pregnancy rate, foaling rate, or other *in vivo* fertility outcomes. Artificial insemination trials are therefore required before conclusions can be drawn regarding reproductive performance.

## 1 Introduction

In domestic animal breeding, fertility and pregnancy rate after mating or artificial insemination are major determinants of reproductive efficiency and economic value. Although male fertility is essential, breeding males are often selected primarily for heritable production or performance traits, whereas semen quality may receive less emphasis if it remains above an acceptable threshold. This is particularly relevant in horses, where stallion selection is commonly based on pedigree, conformation, athletic performance, and competition records (1, 2). Nevertheless, successful pregnancy depends not only on mare fertility but also on several stallion-related factors, including semen quality and the ability of spermatozoa to withstand processing and cryopreservation. Stallions also show marked individual variation in semen freezability, and good-quality raw semen does not necessarily guarantee satisfactory post-thaw semen quality (3, 4).

Semen cryopreservation enables global transport, exchange, sale, and genetic banking of valuable stallion genetics, but freezing and thawing inevitably impose substantial stress on spermatozoa. Cryopreservation may damage the plasma-, acrosomal, and mitochondrial membranes, compromise DNA integrity, and impair motility and fertilizing capacity (5–7). The main sources of cryoinjury include ice crystal formation, osmotic and chemical stress related to cryoprotectants, membrane destabilization, mitochondrial dysfunction, and oxidative stress caused by excessive reactive oxygen species (8, 9). Recent reviews emphasize that stallion sperm cryodamage is multifactorial and highly stallion-dependent (4, 10). Current studies also indicate that interventions targeting oxidative and mitochondrial stress may influence post-thaw sperm function, although their effects are often concentration-dependent and may vary among stallions (11, 12).

Most cryopreservation strategies aim to reduce external stress by optimizing extenders, cryoprotectants, cooling and freezing rates, centrifugation, sperm selection, and thawing procedures. A complementary approach is to increase the intrinsic stress tolerance of the cells before cryopreservation. This concept is based on the biological principle that exposure to a controlled, mild, and reversible stressor can activate endogenous protective mechanisms and thereby improve resistance to a subsequent, more severe challenge. The theoretical background of this adaptive stress response historically originates from the work of Cannon (13) and Selye (14, 15), who described general features of the stress response and demonstrated that moderate stress may induce increased tolerance to a forthcoming, even different type of stressor.

This adaptive response has been tested and applied in several assisted reproduction and *in vitro* systems. Biological materials such as oocytes, embryos, and spermatozoa are exposed to unavoidable stress during *in vitro* fertilization, embryo culture, cryopreservation, semen processing, and artificial insemination. If a controlled preconditioning stimulus is applied, cells activate protective pathways that may improve their survival and functional competence during subsequent procedures. The preconditioning method in this study utilizes a controlled hydrostatic pressure treatment as a mild environmental stressor to enhance cellular tolerance.

As reviewed by Pribenszky et al. (16–18), mild stress preconditioning approaches have shown beneficial effects on gametes and embryos of several species, including bovine, porcine, murine, ovine, and zebrafish models (19). These effects included improved cell survival, pregnancy rate, and litter size. In semen, hydrostatic pressure preconditioning has been shown to improve fertility and offspring number in a porcine model and to improve selected post-thaw quality parameters in boar semen when the treatment parameters and timing were appropriately adjusted (20, 21). These findings suggest that pressure-induced preconditioning may be a useful strategy for improving cryosurvival, but they also indicate that the optimal treatment conditions are species-, cell type-, and protocol-specific. Therefore, mild stress preconditioning parameters must be adapted to the biological characteristics and stress tolerance of the target biological material.

In the present study, we inserted mild hydrostatic-pressure preconditioning into the stallion semen cryopreservation process. We hypothesized that this treatment could improve the post-thaw motility of frozen stallion semen.

## 2 Method

### 2.1 Animals and semen collection

A total of 63 semen samples were obtained from ten clinically healthy stallions (five purebred Arabian and five Shagya-Arabian; age: 14.3 ± 5.6 years) kept at Bábolna National Stud Farm, Hungary. The stallions were housed individually, fed a standard diet, and provided with *ad libitum* access to water. The study was conducted between July and April and was integrated into 31 routine semen collection days at the stud farm. One to four stallions contributed samples on any given collection day, with each sample representing material from a single stallion on a single day. The number of samples collected per stallion was independent of the expected sample quality. Sample distribution across experimental groups is shown in Table 1.

**Table 1:** Sample size, mean, and standard deviation (SD) of total (TM) and progressive motility (PM) of native, post-thaw control, and post-thaw pressure-preconditioned stallion semen samples. Motility values are expressed as percentages.

Treatment group	Sample size (*n*)	TM (%)		PM (%)	
		Mean	SD	Mean	SD
Initial motility	63	75.69	12.27	67.59	13.50
Control	63	51.87	18.08	39.56	17.45
5 MPa/5 min	45	56.79	15.45	44.18	16.22
5 MPa/10 min	63	59.17	19.39	46.90	19.88
5 MPa/30 min	60	58.30	17.98	47.00	18.81
10 MPa/10 min	62	57.06	18.68	46.17	18.95
10 MPa/30 min	60	58.44	16.41	44.94	17.34
20 MPa/10 min	63	56.39	17.23	43.81	17.35

All experimental rounds were connected to routine semen collection and cryopreservation sessions performed at Bábolna National Stud Farm, for routine semen-quality testing. Bábolna first used each ejaculate for routine recording of semen parameters, and the remaining semen sample was then donated for research purposes. No semen collection was performed solely for the purposes of this experiment.

Prior to semen collection, the stallion's penis was thoroughly washed with warm water (42 °C). Ejaculates were obtained after the stallion mounted the breeding phantom. Ejaculates were collected with the help of a Colorado-type artificial vagina, filled with warm water to reach an internal temperature of 42–48 °C according to the stallion's individual preference. Pre-warmed collection bottles equipped with a gel filter were used, shielded with an insulated protective cover.

### 2.2 Semen preparation

After semen collection, initial sperm concentration, total motility, and progressive motility were recorded; these initial values represented sample-level baseline characteristics common to all aliquots derived from the same semen sample. The semen was then extended with centrifugation medium (BotuSemen, Nidacon, Sweden) at a 1:1 ratio. The samples were centrifuged at 600 × *g* for 10 min. After centrifugation, the supernatant was removed, and the sperm pellets were resuspended in freezing extender (BotuCrio, Nidacon, Sweden) and adjusted to 200 × 10^6^ sperm/mL.

Each semen sample was then divided into seven equal aliquots, consisting of one control and six hydrostatic-pressure-treated samples. Each aliquot was aspirated into a separate 2-mL Luer-lock syringe (B. Braun Melsungen AG, Melsungen, Germany) without air bubbles, and sealed with a plastic Luer-lock cap (B. Braun Melsungen AG, Melsungen, Germany). All seven aliquots originating from the same semen sample were processed in parallel. The number of samples per experimental group is detailed in Table 1.

### 2.3 Mild hydrostatic pressure treatment

The hermetically sealed syringes containing the six aliquots were placed individually into the pressure chambers of six manually operated devices (Figure 1), allowing the treatments to be performed in parallel. Before treatment, the pressure chambers were filled with room-temperature distilled water, which served as the pressure-transmitting medium. Pressure was increased to the desired magnitude by tightening the screws of the device.

**Figure 1:**
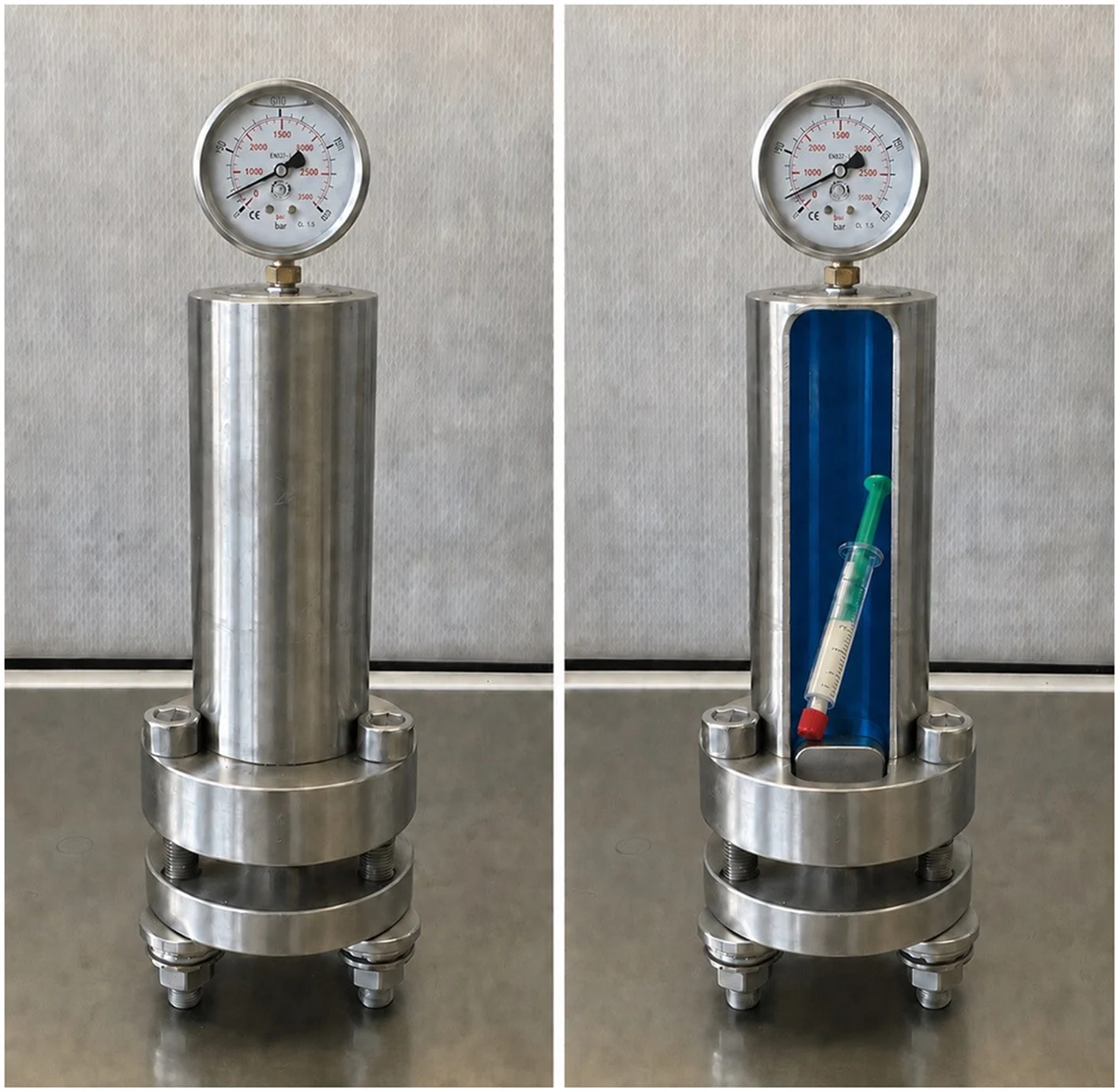
Photo and cross section of the manually operated pressure device. Hermetically closed syringes containing sample aliquots were placed into the water-filled pressure chambers, and the desired pressure was applied by tightening the screws. Six of these were used in the experiment to execute the different levels of hydrostatic pressure impact simultaneously.

Three pressure magnitudes (5, 10, and 20 MPa) and three treatment durations (5, 10, and 30 min) were evaluated within a predefined low-to-moderate pressure range. Six pressure-duration combinations were selected for the split-sample experiment: 5 MPa/5 min, 5 MPa/10 min, 5 MPa/30 min, 10 MPa/10 min, 10 MPa/30 min, and 20 MPa/10 min. The matrix was designed to include a mild short exposure, two longer low-pressure exposures, two intermediate-pressure exposures, and one higher-pressure exposure. These conditions were selected based on previous hydrostatic pressure preconditioning studies in gametes and embryos, previous semen studies in other species (16, 17), and earlier indications that equine spermatozoa may be relatively sensitive to pressure treatment. The aim was to identify effective conditions within this predefined range rather than to perform a full response-surface optimization.

While treatment samples underwent hydrostatic pressure preconditioning, control syringes were kept outside the devices at room temperature for the same duration (Figure 2).

**Figure 2:**
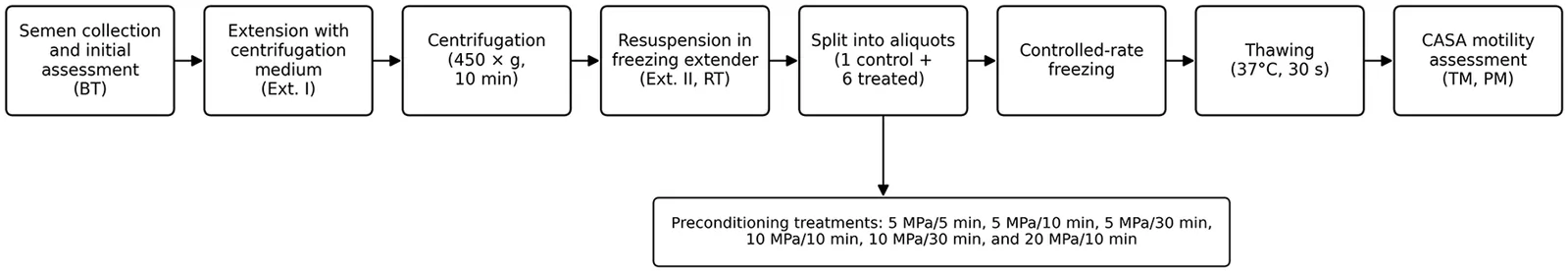
Experimental design. Semen samples obtained during routine collection were extended, centrifuged, resuspended in freezing extender, and divided into seven aliquots (one control and six treated with mild hydrostatic pressure); these were then frozen using a controlled-rate protocol, thawed, and evaluated by CASA. BT, body temperature; RT, room temperature; Ext. I, centrifugation medium; Ext. II, freezing extender; TM, total motility; PM, progressive motility.

### 2.4 Freezing

After completion of the hydrostatic pressure treatments, semen aliquots were emptied into 2-mL Eppendorf tubes from the syringes and aspirated into 0.5-mL artificial insemination straws (IMV Technologies, L'Aigle, France). Two straws were filled for each aliquot (syringe). The straws were sealed using an ultrasonic straw sealer (Ultraseal 21, MTG GmbH, Bruckberg, Germany), placed into a programmable freezer (IceCube 14S-A, SY-LAB Geräte GmbH, Neupurkersdorf, Austria), and gradually cooled to −160 °C according to the manufacturer's recommended freezing protocol. After freezing, straws were stored in liquid nitrogen for 2–8 weeks and subsequently transported to the laboratory to be thawed and evaluated in batches.

### 2.5 Thawing and motility evaluation

For thawing, the straws were placed in a 37 °C water bath for 30 s, after which the semen was transferred into preheated Eppendorf tubes. Motility was evaluated by phase-contrast microscopy using a Nikon Eclipse E200 microscope (Nikon Corporation, Tokyo, Japan) equipped with a heated stage. Motility parameters were measured using a computer-assisted sperm analyser system (AndroVision, Minitube GmbH, Tiefenbach, Germany). For each sample, CASA analysis was performed on at least ten randomly selected microscopic fields and a minimum of 1,000 spermatozoa were evaluated per sample. Total motility and progressive motility were defined according to the manufacturer's guidelines.

### 2.6 Statistical analysis

Total motility (TM) and progressive motility (PM) values were expressed as percentages and transformed to logits before statistical analysis using the following equations: logitTM = ln[TM/(100 – TM)] and logitPM = ln[PM/(100 – PM)]. The transformed values were analyzed using linear mixed-effects models in R statistical software, version 4.4.2, with the lme function of the nlme package (22).

Treatment and season were included as fixed effects, stallion and semen sample nested within stallion were included as random effects to account for the repeated samples taken from the same stallion, and the repeated evaluation of aliquots originating from the same sample. Season had three categories: spring, autumn, and “other”. The “other” category was assigned to two samples collected in July. All pressure-preconditioned groups were compared with the control group using Dunnett's multiple comparison test separately for TM and PM. Because TM and PM represent two related motility outcomes, we additionally evaluated the robustness of the conclusions using a Holm-Bonferroni correction across the 12 treatment-outcome comparisons. This additional correction did not change the interpretation of the results. Estimated odds ratios and 95% confidence intervals were calculated by exponentiating the corresponding model contrasts. Dunnett-adjusted *p*-values < 0.05 were considered statistically significant.

Due to the limited number of stallions per breed (*n* = 5), breed was not included as a factor in the statistical analysis.

## 3 Results

Initial semen quality was recorded for all samples before aliquot division. As shown in Table 1, the overall initial total motility was 75.69 ± 12.27% and the overall initial progressive motility was 67.59 ± 13.50% (mean ± SD).

Post-thaw total motility (TM) and progressive motility (PM) of the six treated groups were compared with those of the control group. Descriptive mean TM and PM values are presented in Table 1, and estimated odds ratios with 95% confidence intervals and Dunnett-adjusted *p*-values are presented in Table 2.

**Table 2:** Estimated odds ratios for total motility (TM) and progressive motility (PM).

Treatment group	TM odds ratio			PM odds ratio		
	Median	95% CI	*p*-value	Median	95% CI	*p*-value
5 MPa/5 min vs. CTRL	1.20	0.991.46	0.078	1.15	0.94–1.40	0.295
5 MPa/10 min vs. CTRL	1.46	1.23–1.74	< 0.001 ^***^	1.41	1.18–1.69	< 0.001 ^***^
5 MPa/30 min vs. CTRL	1.37	1.14–1.63	< 0.001 ^***^	1.40	1.16–1.68	< 0.001 ^***^
10 MPa/10 min vs. CTRL	1.35	1.13–1.61	< 0.001 ^***^	1.39	1.16–1.67	< 0.001 ^***^
10 MPa/30 min vs. CTRL	1.35	1.13–1.62	< 0.001 ^***^	1.25	1.04–1.50	0.009 ^**^
20 MPa/10 min vs. CTRL	1.31	1.10–1.56	< 0.001 ^***^	1.29	1.08–1.54	0.002 ^**^
Spring vs. Autumn	1.62	0.97–2.72	0.067	1.90	1.10–3.25	0.021 ^*^

The first six rows show mild-stress-preconditioned groups compared with the control (CTRL) after freezing and thawing. The final row shows the effect of season on initial motility in spring compared with autumn. Values are shown as odds ratio estimates with 95% confidence intervals and *p*-values. Treatment groups were compared with the control using Dunnett's multiple comparison test. Significance levels: ^*^p < 0.05, ^**^p < 0.01, ^***^p < 0.001.

Mean TM was significantly higher in the 5 MPa/10 min, 5 MPa/30 min, 10 MPa/10 min, 10 MPa/30 min, and 20 MPa/10 min treatment groups than in the control group (56.39–59.17% vs. 51.87%; *p* < 0.001). The 5 MPa/5 min treatment resulted in a numerically higher TM value than the control, but the difference was not statistically significant (56.79% vs. 51.87%; *p* = 0.078).

Mean PM was significantly higher in the 5 MPa/10 min, 5 MPa/30 min, and 10 MPa/10 min groups than in the control group (46.17–47.00% vs. 39.56%; *p* < 0.001). PM was also significantly higher after the 10 MPa/30 min and 20 MPa/10 min treatments (44.94% and 43.81%, respectively; *p* = 0.009 and *p* = 0.002). The 5 MPa/5 min treatment did not significantly affect PM (44.18% vs. 39.56%; *p* = 0.295).

No significant difference was observed (*p* = 0.067) in initial TM between samples collected in spring (March–April, *n* = 23) and autumn (September–December, *n* = 38). However, initial PM was significantly higher (*p* < 0.05) in spring samples than in autumn samples (Table 2). Two samples collected in July were excluded from these comparisons. Although initial motility parameters may have been influenced by season, no significant difference was found in the effect of the preconditioning treatments on TM and PM between spring and autumn samples.

## 4 Discussion

Computer-assisted sperm analyser (CASA) provides an objective and precise method for quantifying sperm motility and is widely used in routine stallion semen evaluation and cryopreservation research. Although post-thaw motility alone does not fully describe sperm function or reliably predict fertility, total motility (TM) and progressive motility (PM) are informative indicators of the ability of spermatozoa to withstand the stresses of freezing and thawing when measurements are performed under standardized conditions (23, 24). Therefore, in the present study, CASA-derived TM and PM were selected as the primary endpoints for comparing paired mild-stress-treated and control aliquots. As a result, mild stress preconditioning treatment significantly increased both post-thaw total motility and progressive motility in frozen-thawed stallion semen compared with the control. Although semen motility may be influenced by season (25, 26), the beneficial effect of mild hydrostatic-pressure treatment was observed independently of the time of year. While motility can be indicative to fertility in mares (27), this motility-based assessment does not replace broader functional testing. Future studies should include plasma membrane integrity, acrosome status, mitochondrial function, DNA fragmentation, capacitation-related parameters, and fertility outcomes to determine whether the observed motility benefit reflects broader improvement in sperm function.

Several other strategies have been investigated to improve stallion semen cryosurvival, including antioxidant supplementation, modification of extenders and cryoprotectants, optimization of centrifugation and cooling protocols, and sperm-selection procedures. The present study did not directly compare pressure preconditioning with these interventions, nor did it test combined treatments such as pressure preconditioning plus antioxidant supplementation. Therefore, the results should be interpreted as demonstrating the benefit of pressure preconditioning relative to paired controls under the cryopreservation conditions used here. Future studies should compare pressure preconditioning with other cryoprotective interventions and evaluate whether combined approaches provide synergistic effects.

In agreement with our results, previous studies reported beneficial effects of mild hydrostatic pressure treatment on sperm motility after cooled storage (28) or cryopreservation (20, 29) of porcine spermatozoa, and cryopreservation of bull semen (30). However, when chilled or frozen-thawed semen is used for artificial insemination, the most relevant biological endpoint is improved fertility, reflected by pregnancy rate, foaling rate, or other reproductive outcomes. The present study did not evaluate artificial insemination using mild-stress-treated or untreated frozen-thawed stallion semen; therefore, our conclusions are limited to post-thaw motility parameters. Nevertheless, previous studies in other species have shown that mild stress preconditioning may improve not only *in vitro* sperm-quality parameters, but also *in vivo* fertility outcomes, including pregnancy rate and litter size, when incorporated into semen-processing or cryopreservation protocols (20, 28, 31). Beyond spermatozoa, mild stress preconditioning has also been investigated in several other reproductive cell types. Beneficial effects have been reported in *in vivo*-produced murine blastocysts, *in vitro*-produced bovine and ovine blastocysts, and *in vivo*-produced zebrafish embryos, with improved survival after conventional freezing or vitrification. Mild hydrostatic pressure treatment of porcine oocytes has also been reported to improve *in vitro* maturation, *in vitro* embryo production, cryotolerance, and outcomes in somatic cell nuclear transfer experiments (16–19).

Despite several successful experimental applications, the biological mechanisms underlying the beneficial effects of mild stress preconditioning are still not fully understood. In embryo and blastocyst models, these effects are frequently linked to the altered expression of stress-response, metabolic, and survival genes (31–34). Although spermatozoa are generally considered transcriptionally inactive cells, mild stress preconditioning has been associated with an increased abundance of specific proteins, namely ubiquinol-cytochrome c reductase complex core protein 1, perilipin, and carbohydrate-binding protein AWN precursor, which may play a role in improved fertilization rates (28). As previously described in other cell types, such changes may result from post-transcriptional stabilization of mRNA or preserved protein conformation induced by mild stress (35).

Other forms of controlled environmental stress, including osmotic (36, 37), oxidative (38) and mechanic stress (39, 40), may also be used to activate endogenous protective mechanisms. However, pressure-based preconditioning has several practical and biological advantages. First, hydrostatic pressure acts instantly and uniformly throughout the sample, without penetration limitations or concentration gradients. Second, hydrostatic pressure preconditioning can be applied with high precision, consistency, reproducibility, and safety using laboratory pressure devices designed for this purpose. In addition, many cell types tolerate relatively broad pressure ranges, giving the method a wide experimental safety margin when appropriately optimized (18). For example, oocytes can survive and retain function after treatment at 20 MPa, and the most beneficial pressure range has been reported to be approximately 10–20 MPa for 30–60 min. Blastocyst-stage embryos appear to have even higher pressure tolerance, with optimal treatment parameters reported in the range of 40–60 MPa for 30–70 min and stress-tolerance levels extending up to 80–90 MPa (17). For spermatozoa, the optimal pressure magnitude has often been reported in the range of 20–30 MPa in several species. However, equine spermatozoa appear to be more sensitive to hydrostatic pressure. Earlier data suggested that treatment levels above 15 MPa before cryopreservation did not produce a significant beneficial effect (41). In the present study, the most favorable responses were observed after treatment at 5–10 MPa, suggesting that lower pressure magnitudes may be more appropriate for stallion semen.

From a practical perspective, hydrostatic pressure preconditioning can be inserted between the semen preparation and freezing steps without changing the subsequent cryopreservation and thawing parts. The treatment is reagent-free and, in the present study, could be performed in parallel with several manually operated pressure chambers. Training requirement is minimal: adjusting the desired pressure range is fast and simple by tightening/releasing the screws, and accurate due to the validated pressure gauge. One device can handle up to 3 ejaculates. Although throughput, commercial implementation, and automation were not thoroughly evaluated, substantial experience has been collected regarding usability. Based on the experience, semen preparation, aspirating into the syringe, de-airing and closing the syringe with a Luer-lock cap took less than a minute. Placing the syringe into the pressure chamber and reaching the desired pressure magnitude by tightening the screws on the side of the chamber also took less than a minute, as did lowering the pressure and removing the sample. Automation of such a procedure with scale-up options is possible. For routine commercial use, standardized laboratory pressure devices and validated operating procedures would be required. Higher-throughput multi-chamber systems and automated devices may further improve workflow integration by reducing operator dependence and enabling simultaneous treatment of multiple samples under controlled pressure and time settings. Therefore, although the present workflow demonstrates feasibility, implementation in commercial equine semen laboratories will require additional validation of throughput, reproducibility, cost-effectiveness, and operator training requirements.

Previous studies indicate that optimal hydrostatic pressure preconditioning parameters are species- and cell-type-specific. Therefore, the first step in developing a mild stress preconditioning protocol is to identify the pressure magnitudes and treatment durations that are tolerated by the target cells without causing irreversible damage. Subsequently, pressure-duration combinations can be tested in two phases. In the first phase, pressure treatment is applied without any further manipulation in order to evaluate its direct effect on cell structure and function. In the second phase, the relevant assisted reproductive procedure, such as cryopreservation, is performed after pressure treatment. In this phase, the expected effect is improved cellular resistance to the subsequent procedure compared with control samples, allowing identification of the most beneficial treatment parameters. Based on the present data, the mildest treatment, 5 MPa for 5 min, appeared insufficient to induce a robust beneficial effect. Although this treatment caused a numerical increase in total motility, the increase did not reach statistical significance, and progressive motility was not significantly improved. At the higher end of the tested range, 10 MPa for 30 min and 20 MPa for 10 min still produced significant improvements, but the estimated effect was smaller than that observed after the most effective treatments. The most favorable responses were obtained after 5 MPa for 10 min, 5 MPa for 30 min, and 10 MPa for 10 min, which significantly increased both total and progressive motility. These findings indicate that mild hydrostatic pressure treatment may be a promising preconditioning tool for improving the post-thaw motility of frozen stallion semen. However, further studies including additional sperm-quality parameters and fertility trials after artificial insemination are required before its practical value in equine reproduction can be confirmed.

The present study has several limitations. First, the pressure treatments were performed using manually operated laboratory devices. Although this setup allowed parallel treatment of the six pressure-duration combinations under controlled experimental conditions, it was not designed to evaluate high-throughput commercial implementation. Second, the tested pressure-duration matrix was selected to cover a biologically plausible low-to-moderate pressure range based on earlier pressure-preconditioning studies and preliminary experience, but the study was not a full response-surface optimization. Therefore, the identified favorable treatments should be interpreted as effective conditions within the tested range, rather than as definitive optimum. Third, the study focused on CASA-derived total and progressive motility and did not include morphology, morphometry, membrane integrity, acrosome status, mitochondrial function, DNA fragmentation, capacitation status, or fertility outcomes. Consequently, the results demonstrate improved motility-based cryosurvival, but they do not prove improved fertilizing capacity or foaling rate. Further studies including broader sperm-quality assessments and artificial insemination trials are required to confirm the practical reproductive value of the treatment.

## Data Availability

The raw data supporting the conclusions of this article will be made available by the authors, without undue reservation.
